# When Big Data Backfires: The Impact of a Perceived Privacy Breach by Pharmaceutical E-Retailers on Customer Boycott Intention in China

**DOI:** 10.3390/ijerph19084831

**Published:** 2022-04-15

**Authors:** Rong Liu, Jiawei Yang, Jifei Wu

**Affiliations:** 1 School of Economics & Management, Nanchang University, Nanchang 330031, China; liurong6@mail3.sysu.edu.cn (R.L.); y2362146000@gmail.com (J.Y.); 2 School of Marxism, Sun Yat-Sen University, No. 135, Xingang Xi Road, Guangzhou 510275, China

**Keywords:** perceived privacy breach, psychological contract violation, customer boycott intention, customer previous trust, pharmaceutical e-retailers

## Abstract

The objective of this study was to explore the impact of a perceived privacy breach by pharmaceutical e-retailers on customer boycott intention, especially the mediating role of emotional violation and the moderating effect of customer previous trust. Data were collected via a questionnaire survey of 335 customers of pharmaceutical e-retailers from China. Our research results showed that a perceived privacy breach by a pharmaceutical e-retailer had no direct effect on customer boycott intention; a perceived privacy breach positively affected emotional violation; emotional violation led to customer boycott intention; emotional violation played a mediating role in the relationship between a perceived privacy breach and customer boycott intention; and customer previous trust positively moderated the mediating effect of emotional violation.

## 1. Introduction

With the rapid development of big data technologies, more and more pharmaceutical e-retailers are applying these technologies to their marketing activities. However, the application of big data technologies in online pharmaceutical retailing is a double-edged sword. On the one hand, pharmaceutical e-retailers can collect and utilize a large amount of customer information to carry out personalized marketing and improve performance [1]. On the other hand, the wide application of big data technologies in marketing practice may deepen customers’ privacy concerns [2]. Sometimes, pharmaceutical e-retailers engage in excessive collection, improper storage, and unauthorized use of customer information. During the COVID-19 pandemic, people have increasingly searched for and bought pharmaceutical products online, which has increased the customer data available to pharmaceutical e-retailers and thus made these actions more likely occur. When such actions occur, the customer may perceive that the pharmaceutical e-retailer has breached their own privacy policies [3], which harms the pharmaceutical e-retailer and limits the benefits of big data technology.

Scholars have revealed the consequences of perceived privacy breaches and examined their negative impacts on business performance, customer perception, and customer behavior. In terms of business performance, a perceived privacy breach by a company may lead to a decrease in its market value [4]. From the perspective of customer perception, a perceived privacy breach negatively affects customer satisfaction [5], customer attitudes [6], and customer trust [7]. A customer’s behavior may also be influenced by a perceived privacy breach. Previous research has found that a perceived privacy breach reduces a customer’s purchase intention [8] and word of mouth [5]. It can be seen that existing studies have focused on the effects of perceived privacy breach on positive variables but neglected its influence on customers’ negative responses. In order to improve customer well-being and social welfare, scholars need to understand not only the impacts of a perceived privacy breach on positive variables, but also to uncover and eliminate its impacts on negative customer responses [9].

In the era of social media, customer boycotts against companies are prevalent and profound negative responses of customers [10]. In recent years, the wide adoption of the Internet and social media has facilitated interpersonal communication and the rapid dissemination of customers’ negative sentiments among communities, which has led to frequent customer boycotts [11,12]. Customer boycotts may seriously harm a company’s reputation and sales [13]. According to existing research, customer boycotts are often caused by companies’ misconduct [14]. Thus, as a form of companies’ misconduct [9], does a perceived privacy breach influence customer boycott intention? What is the mediating mechanism between a perceived privacy breach and customer boycott intention? Is there a boundary condition in the mediating effect?

Drawing on the S-O-R model, this study explored the relationships between a perceived privacy breach, emotional violation, and customer boycott intention. The S-O-R model has been widely applied to explain customer behaviors in online contexts: environmental stimuli (stimuli) affect individuals’ cognitive and affective states (organism), which in turn trigger their behavioral responses (responses), such as approach or avoidance behaviors [15,16]. This study considers a perceived privacy breach as a stimulus (S) in the online pharmaceutical retail environment, emotional violation as an internal state (O), and customer boycott intention as a behavioral response (R). Therefore, combining the S-O-R model and the theory of psychological contract violation, we infer that a perceived privacy breach probably has an impact on emotional violation, which in turn affects customer boycott intention. In addition, we extend the S-O-R model by introducing customer previous trust as a moderating variable in the model.

This study contributes to extant literature in two aspects. First, to the best of our knowledge, we are the first to apply the S-O-R model and the theory of psychological contract violation to the analysis of a perceived privacy breach as well as its consequences in the setting of big data. Second, previous studies have neglected the antecedents and mechanisms of customer boycott intention in online pharmaceutical retailing, and we fill these gaps by examining how customer boycott intention toward pharmaceutical e-retailers is affected by a perceived privacy breach. The findings of this research can provide implications enabling pharmaceutical e-retailers to manage customer information effectively and lower the negative impacts of a perceived privacy breach.

As for the structure of this article, we first evaluate the literature on a perceived privacy breach and customer boycotts before proposing research hypotheses. Then, we undertake a survey to test these hypotheses. Finally, we illuminate the theoretical contributions and practical implications, as well as the limitations of this work and further research topics.

## 2. Theoretical Framework

### 2.1. Literature Review

#### 2.1.1. Perceived Privacy Breach

“Privacy” in this research refers to customers’ information privacy, which in turn refers to the state in which the identity, cognition, behavior, and other private information of customers are not obtained arbitrarily and are effectively protected by pharmaceutical e-retailers [1]. Customers’ information privacy is usually protected according to the privacy policies formulated by pharmaceutical e-retailers, which involve the norms of pharmaceutical e-retailers’ collection, use, and dissemination of customer information [7]. If the customer perceives that pharmaceutical e-retailers have breached these norms, a perceived privacy breach occurs. A perceived privacy breach involves the perceptions of excessive collection [7], improper storage [8], unauthorized secondary use [7], and unauthorized dissemination of information [3]. When a customer perceives a privacy breach by a pharmaceutical e-retailer, they are likely to have negative attitudes [17] and display negative behaviors [9] toward the pharmaceutical e-retailer.

Because customers communicate and interact with pharmaceutical e-retailers via multiple channels, such as by phone or e-mail, pharmaceutical e-retailers can access and utilize a lot of information about their customers with the help of big data technologies, which may lead to a perceived privacy breach [7]. The rapid development of the Internet and big data technologies may bring about a perceived privacy breach in two ways. First, the widespread use of the Internet has created a huge volume of customer information available to pharmaceutical e-retailers, increasing the prevalence of perceived privacy breaches [18]. Second, the advancements of the Internet and big data technologies have made it easier for pharmaceutical e-retailers to obtain and exploit customer information [19], increasing the possibility of a perceived privacy breach. Thus, perceived privacy breaches take place more and more frequently in this contemporary digital era [7] and attract the ongoing attention of researchers.

A perceived privacy breach can lead to many adverse effects. Scholars have focused on three domains of the consequences of a perceived privacy breach: business performance, customer perception, and customer behavior. Regarding business performance, a perceived privacy breach by a company not only decreases its market value, but the negative impact may also spread to the whole industry and reduce the market value of peer companies [4]. As for customer perception, a perceived privacy breach lowers customer satisfaction [5] and can negatively affect customer attitudes by increasing customers’ perceived violation of their expectations [6]. A perceived privacy breach also reduces customer trust and weakens the effectiveness of trust restoration measures, leading to lasting deterioration of customer trust [7]. From the perspective of customer behavior, the perceived unfairness of a perceived privacy breach reduces both the likelihood of a customer promoting the company through word of mouth [5] and the customer’s purchase intention [3]. Moreover, customer behaviors triggered by a perceived privacy breach go far beyond word of mouth and purchase intention. Besides these positive variables (“approach” responses in the S-O-R model), a perceived privacy breach probably leads to negative outcomes (“avoidance” responses in the S-O-R model) [15], which are neglected in existing research. For the sake of customer well-being and company performance, it is important to uncover and eliminate the impact of a perceived privacy breach on customer negative responses.

Customer boycott is a common negative behavioral response of customers to companies’ misconduct [13]. It is reasonable to infer that, as a form of companies’ misconduct [9], a privacy breach probably results in customer boycott intention. Therefore, we attempt to discuss the relationship between a perceived privacy breach by pharmaceutical e-retailers and customer boycott intention in this study.

#### 2.1.2. Customer Boycotts

Customer boycott refers to the action of refraining from buying products of companies owing to their misconducts [13]. Customers usually participate in boycotts by not purchasing products from the boycotted company [20] and switching to products of other companies that comply with ethical or legal norms [11]. Customer boycotts may take place when companies engage in misconduct, such as that relating to environmental damage [13], use of child labor [21], and unethically sourced products [22].

In general, customers may boycott a company mainly because of instrumental and expressive motives. Customers with instrumental motives want to obtain utilitarian benefits through boycotts, such as product returns and economic compensation. Customers with expressive motives want to alleviate their negative emotions and do so by exhibiting them and expressing their values when participating in boycotts [23]. In addition, instrumental and expressive motives differ in the condition of the actors involved. Achieving the goals of instrumental motive-related boycotts often depends on collective pressure toward a company from a group of customers [24]. In contrast, boycotts driven by expressional motives are often initiated by individual customers, who can achieve goals without engaging in collective boycotts [13]. Thus, customer boycotts can involve either the collective actions of a group of customers cooperating to change a company’s practices, or individual actions taken by a single customer for their own emotional ends. We focus on boycotts related to individual customers in this research.

Customer boycotts may harm the relationship between companies and customers [13] and may trigger many negative impacts on companies. It has been revealed previously that customer boycotts degrade company image [20]. In addition, customer boycotts may force companies to change their policies, resulting in a loss of management independence [13]. Boycotts initiated by many customers toward a company may decrease its sales, revenue, cash flow, and stock price [25]. By using the Internet, customers who initiate boycotts are able to contact other customers via social media [26] and persuade them to engage [27], thus expanding the influence of these boycotts.

#### 2.1.3. Psychological Contract Violation

Originating in the field of organizational behavior [28], the term “psychological contract” refers to employees’ perceptions about the mutual commitments between them and the organizations that employ them [29]. Similar to these relationships, psychological contracts can also be established between customers and companies [30]. In the process of interactions with companies, customers gradually form expectations of the companies’ obligations [31]. According to the content of these expectations, there are transactional psychological contracts based on the realization of short-term economic benefits [32] and relational psychological contracts involving long-term social-emotional ties [33]. If customers’ expectations are met, they show more positive attitudes and behavior, such as customer gratitude [34] and recommendations [31]. However, because psychological contracts are subjective and implicit in nature [35], companies and customers often have different understandings of obligations, resulting in companies’ behavior deviating from customers’ expectations [36].

When a company fails to fulfill the obligations expected in the customer’s psychological contract, the customer perceives that the company breaks its promises [37], resulting in a psychological contract breach [38]. “Reneging” and “incongruence” are two common causes of the emergence of a psychological contract breach. “Reneging” refers to a company’s deliberate failure to fulfill their obligations despite making their obligations clear previously. When a company is unwilling to undertake obligations or lacks the ability to do so, this may also cause the customer to perceive a psychological contract breach [39]. “Incongruence” refers to when a customer and a company have different understandings of each other’s obligations. In this case, the psychological contract breach perceived by the customer is still likely to occur even if the company believes that it has fulfilled its obligations [40].

A psychological contract breach may trigger customers’ negative emotions; this is often equated with a “psychological contract violation” [40]. However, a psychological contract breach is different from psychological contract violation. A psychological contract breach is a cognitive result of a company’s failure to fulfill its obligations, whereas a psychological contract violation is an intense negative emotional response that may follow a psychological contract breach [41]. However, it is worth emphasizing that a psychological contract breach does not necessarily lead to a psychological contract violation; this is determined by the nature of the breach and how the consumer interprets the company’s obligations [42]. If a customer attributes the psychological contract breach to reneging and is treated unfairly, a psychological contract violation is more likely to occur [40]. A psychological contract violation can lead to a range of adverse outcomes. In the buyer–supplier relationship, the detrimental impact of unethical buyer behavior on supplier trust is mediated by a psychological contract violation [43]. In addition, a psychological contract violation can reduce customer recommendation behavior [31] and trigger negative word of mouth, boycotts, and other forms of retaliation [38].

### 2.2. Research Hypotheses

#### 2.2.1. Perceived Privacy Breach and Customer Boycott Intention

A perceived privacy breach by a pharmaceutical e-retailer results in customer boycott intention. An important trigger of customer boycott is companies’ misconduct [13]. The main drivers of customer boycott are instrumental and expressive motives [23]. A perceived privacy breach enhances customers’ both instrumental and expressive motives to adopt boycott behavior. A perceived privacy breach can result in customers’ personal information being extensively disseminated on the Internet, increasing the danger of personal information theft and property loss. Consequently, a perceived privacy breach may enhance customer’s instrumental motives, such as wanting to change a pharmaceutical e-retailer’s behavior and receive compensation via boycotts, thus leading to customer boycott intention. Moreover, a perceived privacy breach can trigger negative emotions among customers and drive them to express and alleviate these emotions by refraining from buying products from the company in question [11]. Therefore, a perceived privacy breach may also result in customer boycott intention through customers’ expressive motives. It is thus evident that a perceived privacy breach may strengthen customers’ instrumental and expressive motives, leading to customer boycott intention. Therefore, we propose the following hypothesis:

**Hypothesis** **1** **(H1).** *A perceived privacy breach by a pharmaceutical e-retailer has a positive effect on customer boycott intention.*

#### 2.2.2. Perceived Privacy Breach and Emotional Violation

The S-O-R model assumes that the external environment (stimuli) probably affects the individual’s internal states (organism), such as cognition and emotion [44]. In this study, we consider a perceived privacy breach as an external stimulus (S) and emotional violation as an internal organism (O). Therefore, based on the S-O-R model, a perceived privacy breach by a pharmaceutical e-retailer may trigger emotional violation among customers.

Emotional violation refers to negative emotional experiences resulting from a customer’s perception that a pharmaceutical e-retailer does not respect their privacy and has therefore broken the psychological contract [45]. A principle of the psychological contract between customers and pharmaceutical e-retailers is that pharmaceutical e-retailers should earnestly meet the privacy policy obligations. According to the theory of psychological contract violation, when a pharmaceutical e-retailer infringes upon its privacy policy, the customer perceives that the e-retailer has broken the psychological contract. Furthermore, a psychological contract breach may be triggered by customers’ perception that pharmaceutical e-retailers have reneged on their promise regarding their privacy policies [46], which leads to customers feeling emotionally betrayed, disrespected, and exploited [40] and consequently culminates in emotional violation. Thus, we put forward the following hypothesis:

**Hypothesis** **2** **(H2).** *A perceived privacy breach by a pharmaceutical e-retailer has a positive effect on emotional violation.*

#### 2.2.3. Emotional Violation and Customer Boycott Intention

Drawing on the S-O-R model, an individual’s internal organism (organism) might lead to such individual responses (responses) as approach or avoidance behaviors [47]. In this study, we regard emotional violation as an internal organism (O) and customer boycott intention as a response (R) similar to avoidance behavior. Therefore, based on the S-O-R model, emotional violation probably results in customer boycott intention.

In addition, in light of the theory of psychological contract violation, after perceiving a psychological contract breach and experiencing emotional violation, a customer acts accordingly to cope with the situation [48]. Studies have found customer boycott behavior to be an effective way for customers to ease their negative emotions [11]. When a customer feels negative emotions related to being betrayed by a pharmaceutical e-retailer, they may mitigate the negative impact of emotional violation by engaging in boycott activities [38]. However, customers’ negative emotions can result in unfavorable behavior toward pharmaceutical e-retailers. In the context of a customer’s perception of a privacy breach, it has been revealed that emotional violation motivates negative behavior among customers, such as spreading negative opinions and turning to other brands [45]. Moreover, customers’ negative emotions can also trigger customer boycott behavior [22]. Accordingly, we propose the following hypothesis:

**Hypothesis** **3** **(H3).** *Emotional violation has a positive effect on customer boycott intention.*

#### 2.2.4. Mediating Effect of Emotional Violation

According to the S-O-R model, environmental stimuli (stimuli) affect an individual’s internal states (organism), which in turn triggers behavioral responses (responses). In other words, internal states play a mediating role in the relationship between environmental stimuli and behavioral responses [49]. In this study, we consider a perceived privacy breach as an environmental stimulus (S), emotional violation as an internal state (O) of the individual, and customer boycott intention as a behavioral response (R). Therefore, based on the S-O-R model, a perceived privacy breach and customer boycott intention are mediated by emotional violation.

In addition, considering the theory of psychological contract violation, if a customer believes that their psychological contract with a company has been broken and perceives a psychological contract breach, they may experience emotional violation and thus respond by behaving negatively [50]. Emotional violation mediates the impact of a psychological contract breach on negative behavior [38]. In the setting of online pharmaceutical retailing, a perceived privacy breach by a pharmaceutical e-retailer may break the customer’s belief, founded in the psychological contract, that pharmaceutical e-retailers should earnestly meet the privacy policy obligations. Accordingly, a customer that perceives a psychological contract breach and experiences emotional violation may develop customer boycott intention. Thus, emotional violation acts as a mediator between a perceived privacy breach and customer boycott intention. An empirical study by Martin et al. [45] has shown that emotional violation mediates the influence of customer data vulnerability on customer behavior. Thus, we propose the following hypothesis:

**Hypothesis** **4** **(H4).** *Emotional violation mediates the effect of a perceived privacy breach by a pharmaceutical e-retailer on customer boycott intention.*

#### 2.2.5. The Moderating Effect of Customer Previous Trust

Customer previous trust refers to the extent to which a customer believes a pharmaceutical e-retailer is trustworthy before perceiving a privacy breach. A customer with a higher level of trust is more likely to believe that the pharmaceutical e-retailer keeps its promises in the psychological contract [40]. Customer trust is a reciprocal exchange relationship between a pharmaceutical e-retailer and the customer, in which both parties are expected to offer and gain certain rewards [46]. Therefore, when customer trust toward a pharmaceutical e-retailer increases, the customer has higher expectations that the e-retailer will fulfill its obligations [38]. According to the theory of psychological contract violation, if a customer’s previous trust level is high, a perceived privacy breach makes them experience the psychological contract breach more keenly and elicits a stronger emotional violation [40], thus leading to a more negative behavioral response. Therefore, customer previous trust strengthens the impact of a perceived privacy breach on emotional violation and customer behavior [51].

Specifically, customer previous trust moderates the effect of a perceived privacy breach on customer boycott intention through emotional violation. When customer trust before a perceived privacy breach is high, the influence of the perceived privacy breach on customer boycott intention through emotional violation is stronger than that for a customer with low trust. A customer with higher previous trust tends to actively cooperate with the pharmaceutical e-retailer and have higher expectations that it will fulfill its obligations [38]. Thus, in this situation, a perceived privacy breach results in a stronger negative response [40] and, consequently, customer boycott intention. Therefore, we put forward the moderated mediation hypothesis:

**Hypothesis** **5** **(H5).** *Customer previous trust strengthens the mediating effect of emotional violation on the relationship between a perceived privacy breach and customer boycott intention.*

**Figure 1:**
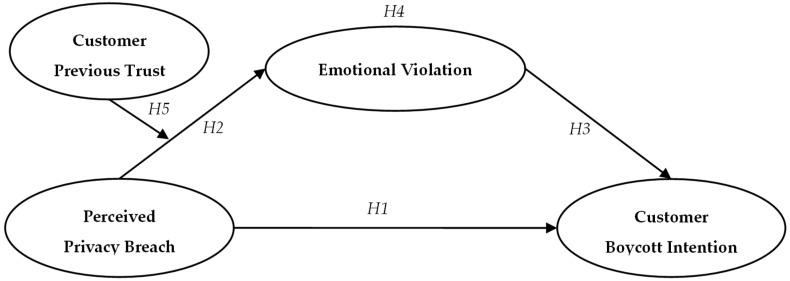
Conceptual model.

To sum up, the conceptual model of this study is shown in Figure 1.

## 3. Methodology

### 3.1. Questionnaire Design and Measures

To test our hypotheses, a survey was employed to collect data. The questionnaire was divided into a warm-up section, the main section, and a basic personal information section. First, the warm-up section asked the respondents to recall an impressive experience of a perceived privacy breach by pharmaceutical e-retailers. Second, the main section consisted of the measures of four key variables: perceived privacy breach, emotional violation, customer previous trust, and customer boycott intention. All these measures were evaluated on a five-point Likert scale (1 = strongly disagree; 5 = strongly agree). Specifically, a perceived privacy breach was assessed by four statements adopted from Bansal et al. [52] and Gerlach et al. [53]. Emotional violation was measured by four statements developed by Martin et al. [45]. Customer previous trust was assessed by three statements used by Martin et al. [45]. The measure of customer boycott intention was designed by Trautwein and Lindenmeier [22]. Finally, the basic personal information section included some control variables, such as the customer’s gender, age, monthly spending, and the history of purchasing that pharmaceutical e-retailer’s products (short for “purchase history” afterwards). Table 1 summarizes the items of the key variables.

### 3.2. Profiling of the Sample

Subjects in this study were customers that have made purchases from pharmaceutical e-retailers. We used Wenjuanxing (www.wjx.cn) (accessed on 1 September 2020), the most frequently used platform for survey in China, to recruit respondents to answer the questionnaires. A total of 380 questionnaires were collected from 1 September 2020 to 29 September 2020, and 45 invalid questionnaires with inattentive answers were deleted, resulting in 335 valid questionnaires. The survey had an effective rate of 88.2%. Females made up 65.4% of the responders, while men made up 34.6%. The bulk of respondents’ ages ranged from 21 to 30 (70.1%). In recent years, young people have become a major group for online shopping in China. In addition, a plurality (84.5%) of respondents had monthly spending of RMB 2000 or less.

## 4. Results

### 4.1. Reliability and Validity

We conducted confirmatory factor analysis (CFA) via AMOS24, and the results are shown in Table 1. The CFA results showed a good fit to the data (χ^2^/df = 1.96, GFI = 0.942, NFI = 0.963, CFI = 0.981, RMSEA = 0.053). Cronbach’s α and composite reliability of the four key variables ranged from 0.88 to 0.95, showing that the reliability was good [54]. Factor loadings of all items were above 0.50, and the values of average variance extracted (AVE) for key variables were from 0.61 to 0.86, revealing strong convergent validity [55].

According to the results of Table 2, the square roots of the AVEs were greater than the corresponding correlation coefficients between the key variables, indicating that the measures had good discriminant validity [56].

### 4.2. Common Method Biases

We assessed the common method biases by conducting Harman’s single-factor test. The results showed that the variance explanation rate of the first factor was 38.68%, which was less than the 50% critical value standard [57], indicating that the common method bias of the data was acceptable.

### 4.3. Hypotheses Test

#### 4.3.1. Direct Effect Analysis

We employed AMOS24 to run the SEM for direct effect analyses (H1, H2, and H3). The results of the SEM are shown in Table 3. The fit indices (χ^2^/df = 1.755, GFI = 0.961, NFI = 0.972, CFI = 0.988, RMSEA = 0.048) indicated appropriateness of the structural model. The coefficients from perceived privacy breach to customer boycott intention (β = 0.023, *p* = 0.788) indicated that a perceived privacy breach had no direct influence on customer boycott intention. Therefore, H1 was not supported. However, a perceived privacy breach had positive influence on emotional violation (β = 0.779, *p* < 0.001), and emotional violation had positive influence on customer boycott intention (β = 0.262, *p* < 0.001), supporting both H2 and H3.

#### 4.3.2. Mediation Analysis

We did the mediating test with SPSS26, using 5000 resampling bootstrapping suggested by Hayes [58]. The result showed that the indirect effects of a perceived privacy breach on customer boycott intention through emotional violation were significant (effect size = 0.160, SE = 0.049, 95% CI (0.071, 0.262)), and the direct effects of a perceived privacy breach on customer boycott intention were not significant (effect size = 0.027, SE = 0.061, 95% CI (−0.094, 0.147)), which meant that emotional violation fully mediated the effect of a perceived privacy breach on customer boycott intention. Thus, H4 was supported.

#### 4.3.3. Moderated Mediation Analysis

To test H5, we followed a bootstrapping procedure based on Hayes’ PROCESS macro, with a confidence level of 95 percent and a bootstrap sample of 5000 [58]. Table 4 shows the results of the analysis. The index of moderated mediation was significant (index = 0.043, SE = 0.022, 95% CI (0.010, 0.096)), indicating that customer previous trust moderated the mediating effects of emotional violation on the relationship between a perceived privacy breach and customer boycott intention. For high-level-trust customers, emotional violation significantly mediated the effect of a perceived privacy breach on customers’ attention (effect size = 0.207, SE = 0.068, 95% CI (0.088, 0.357)). In contrast, for low-level-trust customers, the mediating effect of emotional violation (effect size = 0.105, SE = 0.035, 95% CI (0.047, 0.185)) was still significant but the effect sizes were considerably reduced (attention: from 0.207 to 0.105), in support of H5.

## 5. General Discussions

### 5.1. Conclusions

Drawing on the Stimulus-Organism-Response (S-O-R) model and the theory of psychological contract violation, this study explored the effect of a perceived privacy breach by pharmaceutical e-retailers on customer boycott intention in the context of China. Moreover, the mediating effect of emotional violation and the moderating effect of customer previous trust were investigated. The conclusions of this study are as follows: (1) A perceived privacy breach by pharmaceutical e-retailers had no direct influence on customer boycott intention. (2) A perceived privacy breach by pharmaceutical e-retailers led to emotional violation. (3) Emotional violation resulted in customer boycott intention. (4) Emotional violation played a mediating role in the influence of a perceived privacy breach by pharmaceutical e-retailers on customer boycott intention. (5) Customer previous trust positively moderated the mediating effect of emotional violation on the relationship between a perceived privacy breach and customer boycott intention. When customer previous trust was high, emotional violation played a greater role in mediating the effect of a perceived privacy breach on customer boycott intention than when trust was low.

### 5.2. Theoretical Contributions

The findings of this study have three theoretical contributions. First, this study deepens our understanding of a perceived privacy breach, specifically in the context of online pharmaceutical retailing, and explores the impact of a perceived privacy breach on customer boycott intention and the underlying mechanisms.

Second, this study enriches the research on customer boycotts, which have received increasing attention from marketing researchers and practitioners [22]. This study examines how customer boycott intention is affected by a perceived privacy breach by pharmaceutical e-retailers and its mechanisms, which enriches the research on customer boycotts.

Third, this study extends the application of the theory of psychological contract violation. Until now, scholars have explored the phenomenon of psychological contract violation only in general contexts [38]. This study applies the concept of psychological contract violation to the context of big data marketing and a perceived privacy breach and analyzes the mechanism and boundary conditions of the theory.

### 5.3. Practical Implications

This study has important implications for pharmaceutical e-retailers’ management of customer information. Pharmaceutical e-retailers need to take a holistic view of customer information management. Big data technology allows pharmaceutical e-retailers to collect and use customer information to customize products and promotional strategies to improve customer experience and business performance [3]. However, improper collection and use of customer information can violate privacy policies and harm customer and business interests. This study shows that a perceived privacy breach can make customers experience emotional violation, causing those customers to no longer buy products from one pharmaceutical e-retailer and possibly switch to one of their competitors. Therefore, it is important for pharmaceutical e-retailers to control the collection, use, and management of customer information to avoid adverse effects on business performance.

This study is beneficial for pharmaceutical e-retailers to further understand the role of customer trust when misconduct occurs. In general, pharmaceutical e-retailers believe that a higher level of customer trust is better. However, this study finds that customers with higher levels of trust have stronger negative emotional and behavioral reactions when they recognize that their chosen pharmaceutical e-retailers are engaging in misconduct than those with lower levels of trust. This implies that pharmaceutical e-retailers should carefully handle customers with high previous trust when these customers perceive misconduct.

### 5.4. Limitations and Future Research

Some limitations of this study and opportunities for future research should be considered. First, the data in this study were collected via a survey. Future research should expand the scope of this study by increasing the number of data sources (e.g., experiments, secondary data).

In addition, our findings are based on research in China, and it is difficult to be generalized to other cultural, social, and demographic contexts. Scholars can conduct relevant studies in other countries to validate the findings.

Finally, although this study reveals the mechanism that customers’ perceptions of pharmaceutical e-retailers’ privacy breach influence customer boycott intention, it does not explore the specific actions that pharmaceutical e-retailers can take to mitigate these negative effects. Future research should propose and verify moderating mechanisms that companies can adopt to alleviate such negative effects.

## Figures and Tables

**Table 1:** Measured items and CFA results. α, Cronbach’s α; CR, composite reliability; AVE, average variance extracted.

Variables and Items	Factor Loading	α	CR	AVE
**Perceived privacy breach**	-	0.88	0.88	0.64
I think the pharmaceutical e-retailer collected unnecessary customer information privacy.	0.75			
I think the pharmaceutical e-retailer did not make full efforts to protect customer information privacy.	0.80			
I think the pharmaceutical e-retailer exploited customer information privacy to promote products.	0.84			
I think the pharmaceutical e-retailer shared customer information privacy with other organizations.	0.81			
**Emotional violation**	-	0.95	0.95	0.81
Regarding the pharmaceutical e-retailer’s customer information privacy activities, I feel violated.	0.90			
Regarding the pharmaceutical e-retailer’s customer information privacy activities, I feel betrayed.	0.87			
Regarding the pharmaceutical e-retailer’s customer information privacy activities, I feel not respected.	0.95			
Regarding the pharmaceutical e-retailer’s customer information privacy activities, I feel taken advantage of.	0.89			
**Customer boycott intention**	-	0.82	0.82	0.61
I will not continue to buy the pharmaceutical e-retailer’s products.	0.79			
I would advise others not to buy the pharmaceutical e-retailer’s products.	0.84			
I will switch to buying products from the pharmaceutical e-retailer’s competitors.	0.71			
**Customer previous trust**	-	0.95	0.95	0.86
Before the pharmaceutical e-retailer’s customer information privacy activities, I trusted it.	0.87			
Before the pharmaceutical e-retailer’s customer information privacy activities, it was trustworthy.	0.98			
Before the pharmaceutical e-retailer’s customer information privacy activities, it was reliable.	0.94			
The fitting index result: χ^2^/df = 1.96, GFI = 0.942, NFI = 0.963, CFI = 0.981, RMSEA = 0.053				

**Table 2:** Discriminant validity test. The values in the lower diagonal of the table present the correlations between the constructs, while the values in the diagonal of the table present the square roots of the AVEs of the construct.

Variables	1	2	3	4
1 Perceived privacy breach	**0.80**			
2 Emotional violation	0.60 **	**0.90**		
3 Customer boycott intention	0.20 **	0.30 **	**0.78**	
4 Customer previous trust	0.05	−0.11 *	−0.13 *	**0.93**

N = 335; * *p* < 0.05; ** *p* < 0.01.

**Table 3:** Results of the SEM. PPB: perceived privacy breach; EV: emotional violation; CBI: customer boycott intention.

Hypothesis	β	SE	*p*-Value	Result
H1 PPB → CBI	0.023	0.085	0.788	Not supported
H2 PPB → EV	0.779	0.072	<0.001	Supported
H3 EV → CBI	0.262	0.071	<0.001	Supported

**Table 4:** Analysis results for the moderated mediation effect (*n* = 335). SE, standardized error. Perceived privacy breach as the independent variable, emotional violation as the mediator, customer previous trust as moderators. Confidence interval (CI) is 95%. Bootstrap sample is 5000.

Moderator	Indirect Effect of Emotional Violation				Moderated Meditation Effect			
	Effect Size	SE	LLCI	ULCI	Index	SE	LLCI	ULCI
Customer previous trust (high)	0.207	0.068	0.088	0.357	0.043	0.022	0.010	0.096
Customer previous trust (low)	0.105	0.035	0.047	0.185				

## Data Availability

The dataset used in this research is available upon request from the corresponding author.
